# Using Open Science Tools to Teach Environmental Sciences

**DOI:** 10.1002/ece3.71837

**Published:** 2025-07-22

**Authors:** Mario Zuliani, C. J. Lortie

**Affiliations:** ^1^ Department of Biological Science York University Toronto Ontario Canada

**Keywords:** collaborative learning, education, higher education, open data, open science, science education, teaching

## Abstract

Open science, work and knowledge that are developed in full, offers critical resources that provide students with insights into the process of research in many fields. There are extensive opportunities within environmental sciences to incorporate open science into undergraduate level courses. There are seven major open science concepts that could be used to teach undergraduate environmental science courses that align with professional research activities, including open‐access papers, pre‐prints, open data, open‐source software, published code, collaborative tools for version control, and open notebooks. Here, we assessed the use of these open science concepts in connection to the European Union pillars of open science, outlining key benefits, challenges, and how these tools can be used in undergraduate environmental science courses. Specifically, these tools support a framework for open science structured around eight pillars, providing incentives to collaborate, enhancing transparency and openness, and promoting diversity and inclusivity. Collectively, these tools support teaching environmental science content as many of the skills gained directly relate to analyzing environmental topics and data while supporting transparency to collaborators and stakeholders. This provides learning opportunities including finding and reusing data, team collaboration, and reading and working with code. Further endorsing the use of open science in environmental science courses can enhance these courses as these tools align with professional research activities that are currently being used, including publishing data collected in labs, pre‐print publishing capstone papers or lab reports, openly publishing code used for analysis, and publishing field notes.

## Graphical abstract

Here, we propose the utilization of open science tools to teach upper‐level undergraduate courses. All proposed open science tools support the pillars of open science and can be utilized in various combinations to enhance undergraduate learning.

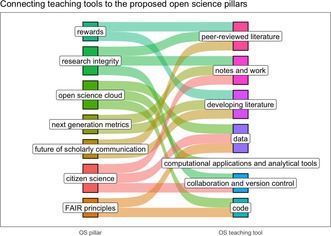

## 1 Introduction

In the current academic landscape, undergraduate students are faced with a learning environment that has significantly and rapidly evolved in the last decade. Students are now expected to adapt to a more digital landscape to navigate these new obstacles (Mollenkopf et al. 2020). Computation is increasingly relevant in all fields of science, particularly in biology and environmental sciences (Markowetz 2017; Hurt et al. 2023). Open science refers to work and knowledge that is developed in full transparency and can exist on a continuum (Vicente‐Saez and Martinez‐Fuentes 2018). Greater emphasis is being placed on open science approaches that improve knowledge sharing, support more rapid iteration, and enable collaborative learning. This shift to open science is occurring with a growing need for experts who have computational and technical skills to work with increasingly large and complex concepts, theories, and datasheets (Kitchin 2014). Thus, upper‐level undergraduate courses have begun promoting open science methods to teach current topics and methods, particularly in the environmental sciences (Harris et al. 2020; Stack Whitney et al. 2022; Pence 2023). More emphasis is placed on the process of conducting scientific studies and their product, while also extending the scope of the work that can be shared beyond paper publications such as data and code (Vicente‐Saez and Martinez‐Fuentes 2018). Hence, there are opportunities to align the learning landscape with its focus on digital tools and learning with the professional landscape in the sciences that also assumes relatively more technical computation skills.

The concept of open science has been proposed as a model through the European Union eight fundamental pillars (Ignat and Ayris 2021). These pillars include FAIR Principles, Research Integrity, Next Generation Metrics, Future of Scholarly Communication, Citizen Science, Education and Skills, Rewards, and European Open Science Cloud (EOS) (Table 1; Ayris 2020). “FAIR principles” refers to research outputs that are findable, accessible, interoperable, and reusable, while also being deposited into trustworthy repositories (Wilkinson et al. 2016; Ayris 2020; Shanahan and Bezuidenhout 2022). The pillar of “research integrity” refers to researchers and published work acting honestly, respectfully, reliably, and are held accountable for their actions while emphasizing accessability (Ayris 2020; Ciubotariu and Bosch 2022). The pillar of “next generation metrics” or “altmetrics,” emphasizes determining the impact research and data have on their respective fields through public outreach, blogging, comments, and annotations (Priem et al. 2012; European Commission Directorate General for Research and Innovation et al. 2017; Ayris 2020). The pillar of the “future of scholarly communication” describes open access publishing for research, methods, and data, promoting the communication of scholarly information that is accessible and inclusive to both academics and the public (Ayris 2020; Baffy et al. 2020). The pillar of “citizen science” primarily focuses upon encouraging public data collection from those not professionally or academically trained and depositing the collected data into repositories such as the Global Biodiversity Information Facility (GBIF) (Groom et al. 2017; Ayris 2020). The pillar of “education and skill” prioritizes open science as a means of teaching and obtaining skills within a respective field (Ayris 2020). The pillar of “reward” emphasizes the concept of rewarding those who actively participate in open science practices (Ayris 2020; Shibayama and Lawson 2021). The final pillar primarily focuses upon endorsing the “European Open Science Cloud” (EOSC), an environment that hosts and processes research data to support and promote European Open Science (Ayris 2020; Almeida et al. 2017). These eight pillars can act as the fundamental framework that structures and supports open science globally, supporting equity and diversity. There are many other frameworks in science that are applicable to teaching undergraduate level courses. For instance, the NASA Transform to Open Science (TOPS) initiative that aims to accelerate scientific discovery, increase the adoption of open science principles, and increase equity and diversity within the scientific community (Ramachandran et al. 2021; Waisberg et al. 2024). Nonetheless, these eight European Union pillars were selected to illustrate how different teaching tools can connect to high level principles and theories (Hampton et al. 2015; Elliott and Resnik 2019; Kohrs et al. 2023).

**TABLE 1:** A summary of representative open science tools that can enhance upper‐level undergraduate courses in the environmental sciences.

Foundation	Foundational concept	Tool	Open science pillar	Application	Benefits	Challenges
1	Peer‐reviewed literature	Open access (OA) papers	Education and skill Future of scholarly communication Rewards Research integrity	Use open access papers for assigned readings as much as possible. Ensure papers represent diversity and breadth of the field. Student applications can include weekly summaries, presentations, and critical analyses. Ensure students observe and comment on accessibility of journal and type of journal (i.e., gold to hybrid offerings)	Articles can be accessed from anywhere globally for free, allowing for remote learning Encourages change in culture and pay models in scientific publishing Set precedent for openness within the scientific community	Publication fees can influence diversity of authors Can limit scope of papers from sometimes more specialized journals
2	Developing literature	Pre‐prints	Education and skill Next generation metrics Future of scholarly communication Rewards	Use pre‐prints to show current topics, concepts, and research being conducted while also illustrating the peer review process. Students can do similar work to peer‐reviewed literature and contrast pre‐prints vs. peer‐reviewed offerings. Students can also explore publishing capstone and papers they produce as pre‐prints	Introduces the peer review process Rapid exposure and introduction to developing research Illustrates future and current work being conducted	Pre‐prints can be variable in quality and extent of development Often papers that are not accepted will not be available if taken out of pre‐print
3	Data	Data published in federated data repositories	Education and skill FAIR principles Open science cloud Citizen science	Query and reuse openly accessible datasets in federated data repositories to teach and conduct symposium style presentations. Assignments to find a dataset from an open repository and conduct independent statistical analysis and data visualization	Students can see a visual representation of data and how it is structured Introduces the concept of data reuse and the challenges associated with it Allow students to evaluate and analyze data from experiments conducted in a wide range of fields that they would otherwise have no way of obtaining data Allow for a higher level of student engagement	Metadata can often be difficult to understand without supporting documentation or publication Can be difficult to harmonize other open data repositories Some data can be beyond the students current understanding of a topic
4	Computational applications and analytical tools	Open source, and freely available software	Education and skill Rewards	Compare and contrast analyses and data handling including import and formatting into different applications. Students can work in different environments and programming languages to better appreciate scripts for reproducible and transparent science	Analysis of previously published dataset with provided or example code Introduces the concept of data reuse and openness	Not all code repositories are publicly accessible Data, metadata, and code can be challenging to understand if not properly organized and formatted for reproduction Understanding the data without prior knowledge could lead to complications Basic understanding of provided coding language and statistics is necessary to understand the information provided
5	code	Published code scripts, snippets, and documented workflows	Education and skill Open science cloud FAIR principles	In statistical courses or in labs with data analysis, students can include code for review. Students can also annotate code to show workflows and document both provenance of data and decisions to manipulate data. Iterations of analyses can also be examined. Assignments can include code, workflows, screenshots, scripts, functions or other appropriate documentation	Introduces the concept of openly analyzing your data to fellow collaborators and yourself in future Illustrates the workflow and decisions made in data handling and analyses Can be extremely beneficial in modifying and reusing code for other purposes similar to what is being used in the example analysis Can illustrate different methods used to clean, filter, analyze, and visualize data that can be reproduced	Understanding the analysis process can be complex if comments and Supporting Information are not provided to illustrate why certain steps were taken Requires training and background on the software, code, and field of study. There is a steep initial learning curve for many tools Not all analyses are published or openly accessible
6	Collaboration and version control	Collaboration tools for provenance, version control, and project management	Education and skill Research integrity Citizen science	Use tools and ecosystems that support version control of uploaded data, code, and supplemental information to aid in illustrating ongoing work. Issues and projects can be shared and developed to manage collaboration. Team science is enabled	Collaboration, project management experience, and importance of team science with tracking of provenance and versions of work are critical to enable replicable science	Several platforms that allow for data, code, and information storage may not have access to version control Can be somewhat difficult to control when multiple individuals are working on the same project at the same time Keeping track of previous versions and determining which contains the information you are looking for can be difficult
7	Notes and the process of science	Open notebooks	Education and skill Citizen science Next generation metrics Research integrity	Explore open‐notebook platforms including Jupyter or Rmarkdown and teach the practice of making the entire process of science open and transparent. Students can explore and apply open notebooks to labs or lectures in courses	Provides a guideline for better experimental design and insights Introduces students to the idea of taking in‐depth observations outside of the data inputted for analysis Typically supported with version control	Can sometimes be difficult to find open science notebooks as they are not as commonly known as other open science tools Notes can be somewhat confusing and cryptic as they are catered to the individuals conducting the experiment Might be difficult to find notes that are specific to a selected study or experiment Not as widely used by the scientific community as open data, open access, and other open science tools

*Note:* Foundational concepts are major themes associated with open science that can be emphasized and used for teaching environmental science courses. Tools are means that can be used to teach foundational concepts in open science. Both benefits and challenges are those that have been identified through teaching environmental science courses and exploring these tools alongside educational literature.

**FIGURE 1:**
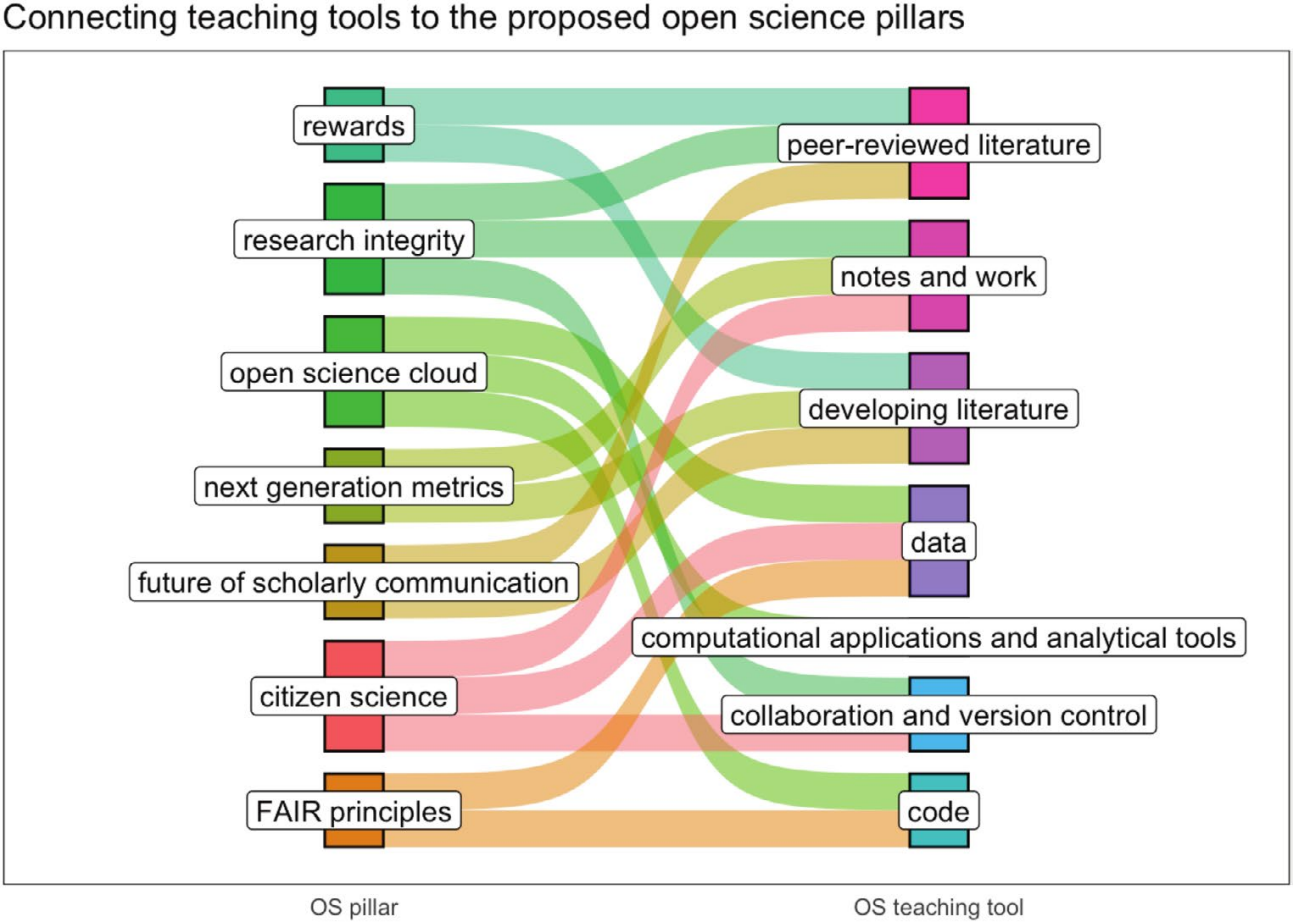
A Sankey plot showing the connection between the proposed European Union open science pillars framework and tested open science tools to support teaching in undergraduate environmental science courses. Colored lines connect each pillar to the relevant teaching tools (although all tools connect to the ‘education and skills’ pillar, see text).

While all pillars of open science play significant roles in the scientific process, the pillar of Education and Skills is crucial as it can shape the culture and practices of the next generation of scientists. Open science is a means to educate and prepare future generations in environmental sciences and can stimulate higher‐level education at the university level. Environmental science is an excellent example to explore tools for teaching, as the practices within this field are relatively open, transparent, and embrace many pillars of open science (Hampton et al. 2015; Hall et al. 2022; Lortie et al. 2023). Within open science education, the foundational concepts can be addressed with corresponding open science tools such as open access peer reviewed literature, open data, and open computational applications and analytic tools (Haaranen and Lehtinen 2015; Scheifele et al. 2021; Dermentzi et al. 2022). Each tool links to a foundational concept and can be used in any combination to teach undergraduate environmental science courses (Jirotka et al. 2013). However, we propose that there are seven foundational concepts that directly relate to the eight pillars of open science, which can support and enhance the undergraduate experience of courses in environmental sciences (Table 1; Figure 1). These concepts and tools are described in detail below, with each instance innovating and supporting the open science fundamental pillars proposed by the European Commission (Almeida et al. 2017).

**FIGURE 2:**
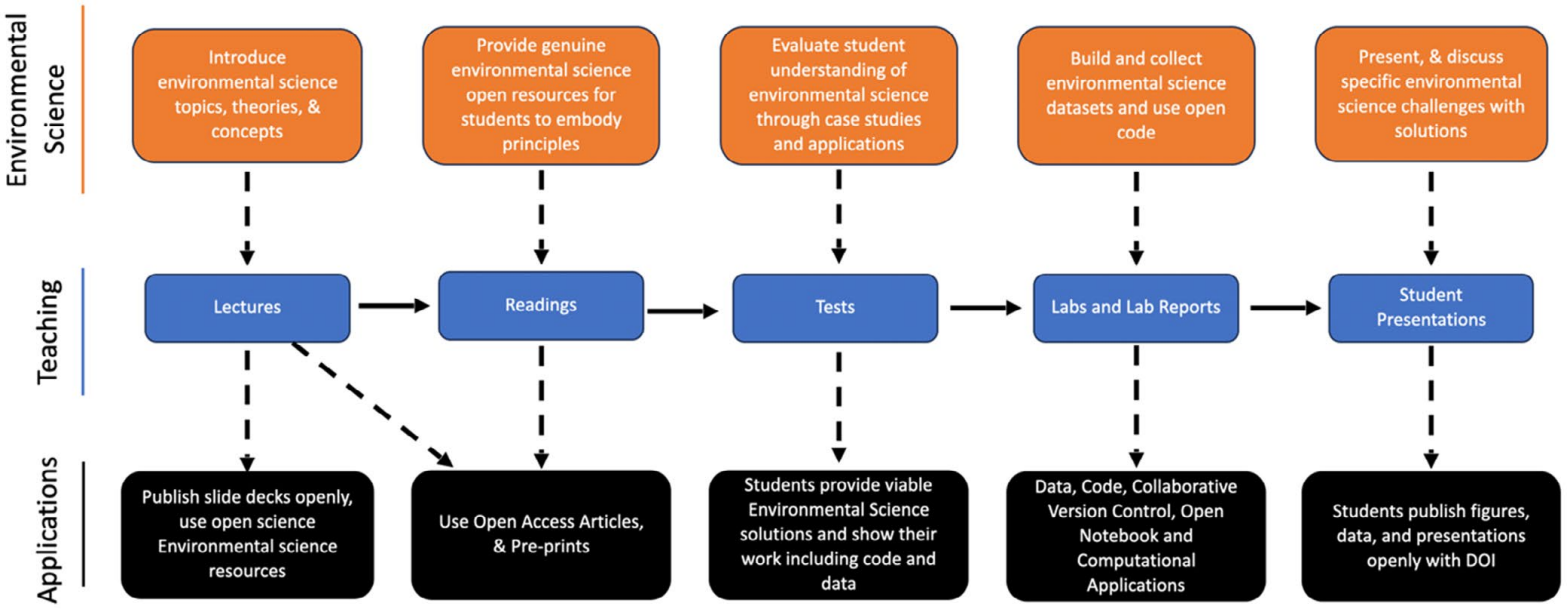
A teaching flowchart showing one set of approaches that can help instructors apply open science tools to specifically enable and connect to environmental science courses. Here we outline common teaching tools that are used in many undergraduate courses. We then show how open science applications can enhance environmental science courses.

Integrating open science tools into environmental science education can transform how students learn and engage with these global issues and challenges. Leveraging open access articles, pre‐prints, and open data, educators can provide students with free access to cutting‐edge research and data. This breaks down barriers to knowledge while providing equitable learning opportunities by making high‐level research unrestricted, allowing for early‐career researchers to readily engage with current research developments, and fosters data literacy and analytic skills without the use of expensive proprietary data sources (Tise and Raju 2013; Giang et al. 2024). These tools provide students with a window into the latest advancements in environmental science, allowing them to access and engage with ongoing debates and evolving research topics—often in real time (Bossu and Heck 2020). By embracing open science tools and practices, educators create more inclusive, dynamic, and hands‐on learning experiences that prepare the next generation of environmental scientists to be critical thinkers, skilled analysts, and collaborative problem solvers in addressing global climate issues (Geange et al. 2021; Mitrano et al. 2023). These open science tools are vital in environmental sciences as researchers need to be up‐to‐date with current theories, while also being proficient in data analysis and modeling to address complex environmental issues (Geange et al. 2021; Mitrano et al. 2023). These tools are not the direct subject taught, but the implicit learning that occurs without acting as the primary subject (Scheifele et al. 2021; Dermentzi et al. 2022). Including any combination of these open science tools into curricula with traditional teaching techniques, including lectures, readings, tests, labs, and student presentations, can enhance the undergraduate learning experience within environmental sciences (Figure 2; Table 2). These tools can better prepare undergraduate students to be technically and computationally adept environmental scientists while also enhancing learning outcome development that educate learners on potential connections and implications of adopting open science tools (Faulconer 2017; Osueke et al. 2018). Environmental science is a critical field that can leverage open science principles and tools as theories and concepts are dynamically changing, while researchers are larger and more complex datasets to address current and future environmental issues (Crain et al. 2014). Promoting the use of these open science practices can help further environmental sciences as many major advancements utilize a combination of these open science tools and concepts (Hernandez et al. 2012; Butts‐Wilmsmeyer et al. 2020).

**TABLE 2:** A summary of open science practices to support teaching environmental science undergraduate courses.

Foundation	Tool	Environmental science applications	Publications	Resources
1	Open access (OA) papers	Careful paper selection for assigned readings in an upper‐year experimental design course, a biology for environmental management course, and a current topics in environmental biology course https://bookdown.org/cj4nature/bio4enviro/	Awareness and attitudes about open access publishing: A glance at generational differences. https://doi.org/10.1016/j.acalib.2014.07.013 Academics' behaviors and attitudes towards open access publishing in scholarly journals. https://asistdl.onlinelibrary.wiley.com/doi/10.1002/asi.23710	Society open access journals including Ecosphere, Ecology and Evolutions, and Environmental Evidence
2	Pre‐prints	Select relevant pre‐print papers as assigned readings in upper‐year environmental management and current topics in environmental Biology Utilize Open Science Framework (OSF) preprint database to select relevant papers https://osf.io/preprints	Preprint articles as a tool for teaching data analysis and scientific communication. https://dx.plos.org/10.1371/journal.pone.0261622 Use of preprint peer review to educate and enculturate science undergraduates. https://onlinelibrary.wiley.com/doi/10.1002/leap.1472	Pre‐print archives including arXiv, bioRxiv, and EcoEvoRxiv
3	Data published in federated data repositories	Carefully devote, conduct, collect, and analyze data from individual experiments in introduction to ecology, and experimental design courses. All data collected by students are publicly published to figshare as groups https://figshare.com/search?q=biol2050 https://figshare.com/search?q=biol3250	Incorporating open data into introductory courses in statistics. https://www.tandfonline.com/doi/full/10.1080/10691898.2019.1669506 Using the problem based learning method and educational technologies to teach open data: A design‐based research approach. https://link.springer.com/10.1007/s10639‐022‐10995‐9	Open data repositories including DataOne, Dryad, Earth BioGenome Project, Global Biodiversity Information Facility, and the Earth System Grid Federation
4	Open source, and freely available software	Biostatistics course explored different analysis and data visualization applications including R, Python, and other ecosystems https://bookdown.org/cj4nature/rstats4bio/	The emergence of GitHub as a collaborative platform for education. https://dl.acm.org/doi/10.1145/2675133.2675284 Student experiences using GitHub in software engineering courses: a case study. https://dl.acm.org/doi/10.1145/2889160.2889195	Open source coding software to analyze environmental science data including R, Python, Quantum GIS, and Geographic Resource Analysis Support System GIS
5	Published code scripts, snippets, and documented workflows	Biostatistics course submitted worked analyses including code and output in R Markdown documents for summative evaluation. https://bookdown.org/cj4nature/rstats4bio/test.html	A practical guide for transparency in psychological science. https://doi.org/10.1525/collabra.158	Using open code to output environmental science analyses including Land Cover Classifications, Soil and Water Assessment Tool (SWAT), Climate Data Analysis, and Distribution Models
6	Collaboration tools for provenance, version control, and project management	GitHub was demonstrated and used as a project management and version control system in a biostatistics course and in a biology for environmental science course https://docs.github.com/en/issues/planning‐and‐tracking‐with‐projects	Teaching git on the side: Version control system as a course platform. https://dl.acm.org/doi/10.1145/2729094.2742608 A database seed for a community‐driven material intensity research platform. https://www.nature.com/articles/s41597‐019‐0021‐x	Using open collaborative tools with version control including Github and Bitbucket
7	Open notebooks	Utilize Jupyter notebook to engage students in open thinking, developing routine notebook use, and to utilize it as a tool for scientific thinking https://jupyter.org/binder https://mybinder.org/provide	Open science notebooks: New insights, new affordances. https://linkinghub.elsevier.com/retrieve/pii/S0378216616306981 Jupyter notebooks as discovery mechanisms for open science: citation practices in the astronomy community. https://ieeexplore.ieee.org/document/8781923/	Using open notebook softwares including Jupyter, R Markdown Notebooks, and Quatro

*Note:* Environmental science applications are specific examples of how to apply open science tools to courses, while publications are representative articles that support or build on these tools and applications. Resources are some examples that can be used for each environmental science application.

## 2 Foundational Concept 1: Open Access Peer‐Reviewed Literature

Peer‐reviewed articles are publicly accessible from open access journals and can be used as an open science tool in teaching undergraduate level courses. Environmental science courses can incorporate multiple articles relevant to course material as a means of illustrating current research, theories, and methodologies. The process of selecting papers published in open access journals will provide more in‐depth analysis and conceptualization (Rodriguez 2014; Rowley et al. 2017). Papers should be selected based on content relevant to lecture material, including those that outline basic definitions, methods, theories, data, models, and critical content (Zhong et al. 2021). However, not every open access journal follows the same publication practices. The current publishing models are changing but now include the following:

1. Gold Open Access: Final version of articles is permanently and freely available to everyone (Dallmeier‐Tiessen et al. 2010).
2. Green Open Access: Articles within a subject‐based or institute‐based repository (Solomon 2013).
3. Diamond Open Access: Journals that allow free access and publication while being supported by institutes or other infrastructure (Fuchs and Sandoval 2013)
4. Hybrid Open Access: Journals that include golden open access articles and impose fees to readers for articles that are not published as open access within the same journal (Björk 2017).

Using any form of open access journal for teaching environmental science can act as an excellent tool providing easier access to material and information globally, while also allowing for more remote and conventional learning. However, Gold and Diamond Open Access journals provide the most accessible versions of peer‐reviewed manuscripts, while Green Open Access is more difficult to locate. In addition, open access journals provide a range of inclusivity depending on the structure of their open access model (Pourret et al. 2022). Models such as Gold and Diamond are more inclusive for authors and readers than subscription or fee‐based open access models, such as the Hybrid Open Access model, as they are full open with no fees for readers (Craig et al. 2007; Pourret et al. 2022).

Introducing the concept of open access publication to undergraduate levels also sets a precedent for openness and collaboration within the environmental science community where knowledge and findings are openly distributed and accessible to everyone (Bartling and Friesike 2014). However, limiting article selection to open access journals could greatly limit the scope of some papers that would otherwise be found in more specialized journals (Rowley et al. 2017). Therefore, teaching with articles published in open access journals provide more opportunities for students to discover current works and topics within environmental science. For example, using open science articles with lecture material can provide a preliminary understanding of environmental concepts while also highlighting current research and methods that have been published. These articles can be used in weekly summaries, presentations, and discussions to further enhance the understanding of these environmental topics. This foundational concept further emphasizes the open science pillar of “future of scholarly communication” because it promotes openness within the academic community by appropriately selecting open access publication models. It is crucial to include open access articles when teaching environmental science because environmental science is rapidly embracing open access publishing with many new and some established journals switching to full open access such as Frontiers in Environmental Science, and Ecology and Evolution (Laakso et al. 2016).

## 3 Foundational Concept 2: Developing Literature

A pre‐print article is a scientific article that is posted to a pre‐print server before formal peer review and publication in a journal (Scheifele et al. 2021). Pre‐print servers, including OSFpreprints (https://osf.io/preprints), arXiv (https://arxiv.org/), and bioRxiv (https://www.biorxiv.org/), can host tens of thousands of potential papers that can be incorporated into teaching undergraduate course readings. For example, using pre‐print servers such as OSFpreprints allow scientists to secure feedback, increase the speed of dissemination, and ensure public access to work (Lee et al. 2019; Moshontz et al. 2021). Specifically, Earth arXiv acts as a pre‐print server specifically for environmental and earth science‐based articles housing publications that are openly accessible from several journals (https://eartharxiv.org/repository/list/). These articles illustrate to students that academia is a dynamic environment with discourse and feedback. Similar to already published articles within open access journals, pre‐prints can act as excellent tools, allowing students to access information remotely and globally. Students can compare pre‐print articles published articles, further highlighting the peer review process and increasing science literacy (McDowell et al. 2022). However, pre‐print articles vary in quality, likely undergoing significant changes during the peer review process (Scheifele et al. 2021). Most pre‐prints that have completed the peer‐review process are likely significantly different from the published manuscript (McDowell et al. 2022). This can be leveraged as a teaching opportunity to show students the peer‐review process in real time while also illustrating the dynamics and versioning of manuscript revisions. This foundational concept can be informative for students intending to pursue research or academic based careers, as it is a window in developing research and illustrate future ideas (Maggio and Fleerackers 2023). To use this tool in an undergraduate environmental science setting, students can for example act as “reviewers” for articles that are currently in pre‐print focusing on environmental issues. This allows students to provide feedback on articles, showing their current understanding of environmental topics while exposing them to the peer‐review process (McDowell et al. 2022). This foundational concept further emphasizes the pillars of “rewards,” “citizen science,” and “next generation metrics” because students can observe academic environment while discovering new research in environmental sciences.

## 4 Foundational Concept 3: Data

Data in the environmental sciences are numerical values, observations, notes, and statistics collected for analysis or reference (Renear et al. 2010; Kempler and Mathews 2017). These data can be qualitative or quantitative, real‐time, or static, and vary in volume (Kempler and Mathews 2017). Open data are any of these forms of evidence that are openly accessible to both academics and the public (Hampton et al. 2013; Gilbert et al. 2018). In recent years, there has been a push for open data to incorporate several scientific fields to conduct analyses that would not have been possible in the past including combining ecological, environmental, and earth sciences (Hampton et al. 2013; Gilbert et al. 2018). Open data are ideally published within federated data repositories, such as DataOne (Murillo 2014) and Dryad (Miller 2016), that allow for easy access to complex raw data analyzed within publications and include detailed meta‐data (Michener et al. 2011). Teaching environmental science with these data not only supports the concept of openness within science, but it also allows students to develop skills with new (and sometimes big) datasets that would typically be inaccessible. For example, students can select specific environmental science‐based data from open data repositories—sites where researchers deposited datasets from original research—conduct their own statistical analysis with the data and present their findings in a symposium style presentation (Rivera et al. 2019; Dermentzi et al. 2022). Teaching environmental science with open data in repositories provides several benefits to undergraduate students including exposing them to large datasets, allowing access to visual representation of data and how it was initially structured, and allowing students to explore a wider range of fields while also highlighting the need to cite datasets in addition to papers (Piwowar and Vision 2013; Zhao et al. 2018; Rivera et al. 2019; Dermentzi et al. 2022). However, one major challenge is that many of these datasets can be very technical or lack proper meta‐data that would allow for replication (Hampton et al. 2013; Zuiderwijk et al. 2012). This can easily be remediated by endorsing the inclusion of detailed meta‐data and notes. Current environmental sciences extensively use open data with many journals requiring data to be openly accessible with published links. There are also major open data projects such as the Earth BioGenome Project (EBP; https://www.earthbiogenome.org/), the Global Biodiversity Information Facility (GBIF; https://www.gbif.org/), and the Earth System Grid Federation (ESGF; https://esgf.llnl.gov/). These projects provide undergraduate students with an opportunity to engage with data collected across multiple fields of disciplines that would otherwise be difficult for them to obtain from labs or researchers. This can enhance student understanding of both the experimental process and analysis of the corresponding data. These skills can be developed using coding exercises, team‐led assignments, and scientific poster presentations to help visualize and analyze data (Toelch and Ostwald 2018; Bakermans 2024). This foundational concept not only encompasses the pillar of “education and skill,” but also the pillars of “open science cloud” and “FAIR principles,” as the data uploaded is easily findable, accessible, interpretable with the appropriate metadata, and reusable. There are other principles that similarly support diversity and inclusion within environmental science, specifically seen through the CARE principles (Collective Benefit, Authority of control, Responsibility, and Ethics) (Carroll et al. 2021). These CARE principles can be used in combination with the FAIR principles of open science enhance accountability, while bringing purpose to resolve Indigenous Peoples' rights to and interests in their data (Carroll et al. 2021). Cloud computing can include RStudio instances online (https://posit.cloud/), application on university servers, data repositories, and even open data retrieved directly from the web (Whaiduzzaman et al. 2014; Banimfreg 2023). It also rewards researchers that have published data through downloads, views, and other usage metrics provided by many repositories (Piwowar and Vision 2013).

## 5 Foundational Concept 4: Computational Applications and Analytic Tools

Open source software and applications are products within public domains with source code that can be read, analyzed, or changed (Bahamdain 2015). These open source applications are freely available computational tools with source code that is freely available and can be redistributed or modified (Bahamdain 2015). Programming languages including R (https://posit.co/downloads/), and Python (https://www.python.org/) all allow for easy and open access to coding software for environmental science data, visualization, and statistics (Ye et al. 2021). These tools provide new opportunities for students to analyze data and code with example materials, while providing the opportunity to write and conduct their own analysis. For example, in combination with open data, students can collect large environmental science datasets, analyze, and visualize their data and can present them as a scientific poster or presentation as a final assignment. Many of these applications are comprised of public “packages” or “libraries” that are created by the community to solve specific challenges (Vuorre and Crump 2021). For example, the open source application R relies on “packages” created by experts in the community to clean, manipulate, and visualize datasets (Vuorre and Crump 2021). Specifically, within environmental sciences there are several applications that can be used as computational applications and analytic tools including Quantum GIS (QGIS; https://www.qgis.org/) and Geographic Resource Analytics Support System GIS (GRASS GIS; https://grass.osgeo.org/). These applications provide the means for students to analyze their own environmental data or previously published datasets that can be acquired from repositories such as Zenodo and Figshare, introducing the concept of data reuse (Zagalsky et al. 2015; Feliciano et al. 2016; Peters et al. 2017). In addition, basic understanding of these applications is required for students to properly use these analytic tools. This foundational concept not only supports the pillar of “education and skills,” but it also supports the pillars of “rewards,” and “open science cloud” as students can openly upload their own analyses, visualization, and statistics to these open access repositories, obtaining dois and displaying their experience in science.

## 6 Foundational Concept 5: Code

In combination with open data and freely accessible and open‐source computational applications, code can be used to analyze and manipulate datasets acquired from open source data. Code or scripts are collections of rules, principles, and regulations intended to run data visualization and statistics (Seale and Kelly 2004; Bell et al. 2017). At the undergraduate level, coding can allow students to analyze datasets by manipulating the data, running statistical analyses, and visualizing specific variables (Bell et al. 2017). However, many environmental science courses do not utilize this tool as many instructors are not well versed in data science skills and research‐based teaching practices (Emery et al. 2021). These skills can be enhanced through the use of published code scripts, snippets, and documented workflows. Within statistical courses and those with labs requiring data analysis, students can include their code for review (Krusche et al. 2016; Indriasari et al. 2020). Coding in environmental science focuses primarily on quantitative data using quantitative data, combining environmental metrics with geospatial information to create predictive models through several methods including Land Cover Classifications, Soil and Water Assessment Tool (SWAT), Climate Data Analysis, and Distribution Models (Clark and Gelfand 2006; Francesconi et al. 2016; Sun and Scanlon 2019). Utilizing these methods in environmental science allows for visualization of critical data through maps, charts, and interactive dashboards, making it more accessible and impactful (Sun and Scanlon 2019; Matheus et al. 2020). Conducting projects with these coding applications also allows for annotation, or comments, of workflows. Annotations are remarks used to support code with metadata or notes on the process (Sulír et al. 2016). These annotations must use the comment tag for the language that the code sample is written in. For example, R uses “#” before inputting metadata, excluding it from the rest of the code (Mendez et al. 2020). This will further illustrate thought processes and decisions to manipulate data. This introduces the concept of collaboration and can be used to illustrate the workflow of example code through the provided annotations and metadata (Light et al. 2014; Klein et al. 2018). However, if those publishing code publicly do not include annotation on their workflow, then understanding and replicating the analysis process can be complex (Filazzola and Lortie 2022). Unfortunately, without prior training and understanding of the software, code, or field of study, there may be significant learning curves (Bernardo and Macht 2021). To improve, courses can be designed to instruct students on how to use these tools, thus, reducing the learning curve or to at least introduce various tools in lectures and labs through examples provided by instructors (Bernardo and Macht 2021). This foundational concept is not entirely different from open source software and analytic tools. By combining coding with other foundational concepts, such as open data and open source applications, undergraduate students can further their understanding of open science as they can utilize a combination of these concepts to analyze and visualize scientific information. This can be used for students to conduct their own analysis on data they acquired from open sources, such as environmental factors that are influencing various processes and species within an area (Emery et al. 2021). Developing these skills early in a researcher's career can further enhance their understanding with environmental datasets while also promoting higher level analyses (Missett et al. 2010; Bakermans 2024). This foundational concept connects with the open science pillars of “European Open Science Cloud,” and “FAIR principles,” as these pillars promote openness and transparency not only in data and publications, but all aspects of the scientific process including analysis and visualization through coding annotations.

## 7 Foundational Concept 6: Collaboration and Version Control

Version control is comprised of multiple layers of a project where differences between submissions are displayed, supporting changes to be potentially undone (Ruparelia 2010). For example, GitHub uses the version control software Git which can be used as one of these project management systems as it allows for version control of all submitted work into a repository (Haaranen and Lehtinen 2015). GitHub provides the opportunity for collaboration through the creation of “Issues,” which are items that can be used to plan, collaborate, and discuss (Bissyande et al. 2013). In addition, GitHub provides the opportunity for version control through deployments, allowing for collaborators to view all previous iterations of a project (Haaranen and Lehtinen 2015). For example, Bitbucket can provide similar capacities for collaboration and version controlling (Chakraborty and Aithal 2022). A key skill for undergraduate students to develop is collaboration through group projects and assignments (LaBeouf et al. 2016; Knox et al. 2019). This concept can also promote “Team Science” which is a collaborative and cross‐disciplinary approach to scientific inquiry through collaborative centers and groups (Bennett et al. 2018). Collaboration with version control allows students to use repositories and applications that allow for version tracking for all potential collaborative assignments. For example, students working on an oral presentation or collaborative assessment can use these version control systems to track group member progress and contributions, while ensuring that previous work is not lost (Cochez et al. 2013). These repositories promote collaboration and versioning, allowing students to enhance their collaboration and communication skills as a team, while multiple individuals work on the same project simultaneously (Heeren and Fishman 2019). Combining version control with open access code further enhances collaboration because students can comment on steps, show their work, and return to previous versions of submitted work (Nüst et al. 2020). Environmental science projects, like those in many fields, rely on data analysis, modeling, and simulations, all of which require reproducibility, replication, and collaboration. Specifically, version control is used in environmental sciences to track changes to models and simulations through systems such as GIT (https://git‐scm.com/), data analysis pipelines to track code used in processing data, and to conduct collaborative research projects across multiple fields such as biology, ecology, and geography (Haaranen and Lehtinen 2015; Heeren and Fishman 2019). This promotes team science, emphasizing the need for collaboration and openness within the scientific community (Bennett et al. 2018). However, keeping track of various versions of a project can become difficult as miscommunication between contributors is likely. It can also be difficult to find the specific information, code, or data within a specified version. However, if used correctly, these collaborative and version control tools can allow students to collaborate on projects, building team‐working skills while promoting open science. This foundational concept promotes the open science pillars of “research integrity”, and “open science cloud,” as it allows other researchers to openly observe the various versions of committed work and/or data, promoting transparency and openness within the scientific community.

## 8 Foundational Concept 7: Open Notes and the Process of Science

Open notes and the process of science can be openly accessible through notebooks illustrating developing comments, concepts, sketches, and ideas used during the process of doing projects and experiments in science (Clinio and Albagli 2017). These notes can be taken and published openly by researchers or by the public. This final foundational concept focuses on open notes to share the process of science for a particular project, experiment, study, or lab. Exploring open notes platforms including Jupyter (https://jupyter.org/), R Markdown notebooks (https://rmarkdown.rstudio.com/lesson‐10.html), quatro (https://quarto.org/), blogs for science, and wikis can be used in teaching undergraduate level students' openness during the scientific process, not just for data, code, or final papers (Bradley 2007). Applications such as Jupyter allow researchers to outline all data that would typically be recorded during the conduction of a project including notes, rough sketches, and basic observations (Wofford et al. 2020). The use of Jupyter can also address Foundational Concepts 3 (Data) and 4 (Code) because this application allows for the inclusion of data and code with narrative text, interactive computations, and visualizations (Wang et al. 2020; Granger and Perez 2021). This shows how various open access tools can be used for multiple Foundational concepts, depending on the specific goals associated with each environmental science course. However, there are many instances in which these notes would be cleaned or more formalized than traditional field notes. Jupyter is an open‐source software and community that builds software, notes, and services that can be used in open science (Wofford et al. 2020; Granger and Perez 2021). This would allow students to gain insight into a researcher's way of thinking through examining field or in lab notes taken during projects and experiments (Carter‐Thomas and Rowley‐Jolivet 2017). In addition, these open notes allow students to collaborate with others on group projects and assignments. Open note applications provide a basic guideline for better experimental design to undergraduate students while also introducing the idea of taking complex in‐depth observations outside of those used in datasets (Kim and Henke 2021). From our previous experience with teaching ecology and evolution, and environmental science courses, students range in their note‐taking abilities with some taking minimal/less descriptive notes, while others take in‐depth/precise notes. Using these open access notes as examples can show students how to take adequate and accurate field notes. However, openly published notes are and can be difficult to find. Attempting to find notes on a specific study or project might prove to be difficult if not impossible as these notes may be unavailable or non‐existent. Science is the process of discovery often through trial and error, making notebooks challenging to decipher as they are catered to the researcher and can be dynamic works‐in‐progress (Simon 1986; Rule et al. 2018). However, these notebooks can promote more openness to a future scientific community and the public through sharing and accessibility of experimental notes (Caprarelli et al. 2023). The concept of open notes can be used in combination with several of the other proposed concepts previously listed, such as open data and code. This foundational concept connects directly with not only the pillar of “education and skills,” but also the pillars of “citizen science,” and “research integrity,” as these notes are open to everyone both within academia and to the public, promoting openness and transparency of the scientific process. Introducing these concepts to students early provides the framework for collaboration in environmental sciences. Specifically, open notes in environmental sciences can ease communication with stakeholders by providing transparency and communicate clear findings, support policy and management decisions, and facilitates environmental monitoring (Barba et al. 2019; Granger and Perez 2021).

## 9 Implications

Open science is a key movement in environmental science promoting transparency within the scientific community and can be used to enhance undergraduate education. The foundational concepts and tools proposed here support both open science research and teaching undergraduate‐level environmental sciences, as they not only build on fundamental skills needed to succeed at the undergraduate level, but also promote skills needed for future careers in many disciplines including the sciences. While there are some challenges associated with the tools used with each concept, we propose that the benefits can enhance the undergraduate learning experience, better preparing students who intend to continue into scientific fields. We propose that these open science tools can be used with more traditional teaching methods in environmental science to not only enhance the understanding of course material, but to enhance critical thinking, analytic, and problem‐solving based skills (Figure 2; Table 2). While these traditional teaching techniques can successfully develop environmental science concepts and theories, here we propose that incorporating these open science tools not only enhances learning but also promotes an equitable learning environment. Many of the tools associated with each concept can be incorporated in environmental sciences, but also in all scientific education, as readings, data, and applications are obtainable in nearly all fields. Most of the typical evaluations in teaching, both formative and summative, can be included in these concepts using our listed tools. However, this is not an exhaustive list, and we recognize that different disciplines have other tools similarly used in science and teaching. Further, there are alternatives to the 8‐pillar EU model and more other core principles of open science to consider. In environmental sciences, the tools and concepts we proposed are increasingly used to communicate and share scientific theories, methodologies, and ideas (Ayris 2020). These tools should be promoted to undergraduate students so that they can develop competitive skills for employment not only in environmental science, but all professional careers, as many of these skills including version control, data, coding, and collaboration are highly transferable. Using these tools and concepts can highlight the eight fundamental pillars of open science thereby promoting openness, accessibility, inclusivity, and diversity within the environmental science community. All the proposed concepts do not need to be included, as emphasis can be placed on specific skills that are more applicable to a course. For example, an upper‐level environmental science course that focuses particularly on experimental design and visualization can focus primarily on data analysis and openness. This may put more emphasis on the fundamental concepts of data, computational applications and data analysis, and code, over other proposed foundational concepts. We encourage the inclusion of these tools and concepts in the current environmental science curricula to endorse openness and collaboration for future scientific generations, promoting further transparency of scientific works (Robson et al. 2021). We recommend incorporating these foundational concepts not only in environmental science courses, but in all undergraduate science courses.

## Author Contributions

**Mario Zuliani:** conceptualization (equal), formal analysis (equal), methodology (equal), project administration (equal), visualization (equal), writing – original draft (lead), writing – review and editing (lead). **C. J. Lortie:** conceptualization (equal), data curation (equal), funding acquisition (lead), methodology (equal), project administration (equal), supervision (lead), validation (equal), visualization (equal), writing – original draft (equal), writing – review and editing (equal).

## Conflicts of Interest

The authors declare no conflicts of interest.

## Data Availability

No new data were created or analyzed in this study.
